# Editorial on “New perspectives in neurosteroids action: a special player allopregnanolone”

**DOI:** 10.3389/fncel.2015.00133

**Published:** 2015-04-09

**Authors:** Valerio Magnaghi, Giulia Puia

**Affiliations:** ^1^Dipartimento di Scienze Farmacologiche e Biomolecolari, Università degli Studi di MilanoMilano, Italy; ^2^Dipartimento di Scienze della Vita, Università degli Studi di Modena e Reggio EmiliaModena, Italy

**Keywords:** steroid, progestagens, non-genomic mechanisms of steroid hormones, neuroprotective agents, pain

The hypothesis that neurosteroids are synthesized and active in the nervous system is well-established among neuroscientists. Their presence was shown for the first time more than 20 years ago (Baulieu and Robel, 1990), however we believe that different neurosteroids actions in central (brain and spinal cord) and peripheral nervous system are not still fully elucidated, deserving further investigation.

Allopregnanolone (Allo) is the most important neurosteroid (Baulieu and Robel, 1990), targeting both neurons and glial cells in central and periphery, primarily through rapid “non-genomic” action via γ-amino butyric acid (GABA) type A receptor (GABA-AR). Given these prerogatives, Allo is a promising drug for development of novel neuroprotective and neuroregenerative strategies, as well as for the treatment of neuropsychiatric disorders. Rupprecht overviewed the state of the art of Allo's actions in the brain, suggesting different molecular mechanisms beyond the GABA-AR interaction. He explores the therapeutic use of Allo and its synthetic analogous ganaxolone in human psychiatric disorders, suggesting that the analysis of neurosteroid profile in neuropsychiatric patients is necessary (Rupprecht, 2014). Ganaxolone has been recently proposed for epilepsy and infantile spasms treatments (Riikonen, 2014), however its use for neuropsychiatric diseases is emerging. The posttraumatic stress disorder (PTSD) is a severe neuropsychiatric condition for which efficient therapies are still lacking. Ganaxolone, acting on GABAergic system, is a therapeutic alternative to the serotonin reuptake inhibitors, the only drugs currently approved by the Food and Drug Administration for PTSD. Pinna and Rasmusson suggested that restoring Allo's brain levels is beneficial in PTSD patients. Using an *in vivo* PSTD mice model, they also demonstrated that the increased corticolimbic levels of Allo reduce the PTSD-like behavior. Therefore, they proposed ganaxolone as alternative treatment for patients suffering PTSD or other disorders implying Allo biosynthesis impairment (Pinna and Rasmusson, 2014).

Allo has recently emerged as neurogenic molecule acting on neural progenitor cells. Interestingly, it may also activate glial cells proliferation and differentiation, in the central or in the peripheral nervous system. Focusing on the neural architecture and neurogenesis in the nigrostriatal tract, Wang proposed Allo as a neurotrophic agent able to stimulate the number of total cells and to re-establish the dopaminergic neurons circuitry (Wang, 2014). This striking approach has been so far suggested to cure neurodegenerative diseases, such as Parkinson and/or Alzheimer. Indeed, Allo reduces β-amyloid protein levels and neuroinflammation, revealing as efficient molecule for the treatment of Alzheimer and other neurologic disorders. Brinton and colleagues presented a safe treatment with Allo that has been optimized for neuroregeneration and reduction of Alzheimer symptoms. Moreover, by tailoring doses/regimen to the different etiologies, Brinton proposes Allo as novel reliable approach for multiple sclerosis, Niemann-Pick, diabetic neuropathy and traumatic brain injury (Irwin et al., 2014). The brain of patients with multiple sclerosis presents a dysregulation in Allo biosynthesis. The hypothesis discussed by Power and colleagues raised the possibility that changes in Allo biosynthesis may control leukocyte functions and the neuroinflammation associated to multiple sclerosis. They found that Allo administration ameliorates neurobehavioral deficits of animals with autoimmune demyelination, proposing its use for neuroinflammatory pathologies (Noorbakhsh et al., 2014).

The pineal gland is still a neglected structure among neuroendocrinologists. Tsutsui and Haraguchi provided clear evidences that the gland is an important neurosteroidogenic organ. They reported a new neuroprotective role of Allo in the brain. During the development, Allo from the pineal gland prevents Purkinje cells death by suppressing the caspase-3 activity (Tsutsui and Haraguchi, 2014).

Neurosteroids are promising drugs also for the treatment of pain. However, they display some side effects such as sedation, amnesia and tolerance, restricting their therapeutic use. Poisbeau, Schumacher and colleagues overviewed the analgesic effects of endogenous neurosteroids, focusing on pharmacologic strategies aimed at stimulating local production of 3-alpha reduced neurosteroids (Poisbeau et al., 2014). This approach limits the side effects, targeting specific structures equipped with the neurosteroid biosynthetic machinery, including the mitochondrial translocation protein complex TSPO. In the perspective of Puia and Magnaghi the functional cross-talk between Allo, the protein kinase type C (PKC) and GABA-AR was discussed. It was reviewed how GABA-AR is modulated by Allo and/or PKC phosphorylation, through molecular mechanisms that can be mutually interconnected (Puia et al., 2015).

To further complicate the Allo's action, the possibility to figure out alternative molecular mechanisms is becoming increasingly credible. A family of new membrane progesterone receptors (mPRs) has been identified as putative target for this neurosteroid. Frye et al. (2014) faced Allo's effects on behavioral processes, involving rapid modulatory actions via GABA-AR and/or n-methyl-D-aspartate (NMDA) receptors. They also characterize the role of promiscuous nuclear receptor, the pregnane xenobiotic receptor (PXR), which may bind Allo in the central nervous system. This mechanism is supposed to support Allo's effects on the midbrain ventral tegmental area (VTA), controlling lordosis and sexual behavior.

In conclusion, Allo is a novel, promising, alternative and reliable drug with several neuroprotective properties. We very much hope this issue will help readers to understand pros and cons of Allo “neuroactions!”

## Conflict of interest statement

The authors declare that the research was conducted in the absence of any commercial or financial relationships that could be construed as a potential conflict of interest.
